# Cadmium as the Critical Limiting Factor in the Co-Disposal of Municipal Solid Waste Incineration Fly Ash in Cement Kilns: Implications for Three-Stage Water Washing Efficiency and Safe Dosage Control

**DOI:** 10.3390/toxics13070593

**Published:** 2025-07-15

**Authors:** Zhonggen Li, Qingfeng Wang, Li Tang, Liangliang Yang, Guangyi Sun

**Affiliations:** 1 School of Resources and Environment, Zunyi Normal College, Zunyi 563002, China; lizhonggencn@126.com; 2 Guizhou Research and Designing Institute of Environmental Sciences, Guiyang 550081, China; litang90426@163.com; 3 Key Laboratory of Karst Georesources and Environment, Guizhou University, Ministry of Education, Guiyang 550025, China; gs.liangliangyang23@gzu.edu.cn; 4 State Key Laboratory of Environmental Geochemistry, Institute of Geochemistry, Chinese Academy of Sciences, Guiyang 550081, China; sunguangyi@mail.gyig.ac.cn

**Keywords:** municipal solid waste incineration fly ash, heavy metal, co-disposal with cement kilns, three-stage water washing, dosage estimation

## Abstract

The co-disposal of municipal solid waste incineration fly ash (MSWI-FA) in cement kilns is an effective method for managing incineration by-products in China. However, the presence of heavy metals in MSWI-FA raises environmental concerns. This study analyzed the Cu, Zn, Cd, Pb, Cr, and Ni concentrations in MSWI-FA from 11 representative facilities across China and assessed the efficacy of a three-stage water washing process for Cl and heavy metal removal. The results revealed significant regional variations in heavy metal content that were strongly correlated with surface soil levels, with Zn, Pb, and Cu exhibiting the highest concentrations. Elemental correlations, such as Cu-Pb and Zn-Cd synergies and Cd-Ni antagonism, suggest common waste sources and temperature-dependent volatilization during incineration. The washing process (solid–liquid ratio = 1:10) achieved 97.1 ± 2.0% Cl removal, reducing residual Cl to 0.45 ± 0.32%, but demonstrated limited heavy metal elimination (10.28–19.38% efficiency), resulting in elevated concentrations (32.5–60.8% increase) due to 43.4 ± 9.2% mass loss. Notably, the washing effluents exceeded municipal wastewater discharge limits by up to 52-fold for Pb and 38-fold for Cd, underscoring the need for advanced effluent treatment. To mitigate environmental risks, the addition of washed MSWI-FA in cement kilns should be restricted to ≤0.5%, with Cd content prioritized in pre-disposal assessments. This study provides actionable insights for optimizing MSWI-FA co-processing while ensuring compliance with ecological safety standards.

## 1. Introduction

Due to urbanization and improvements in living standards, the volume of municipal solid waste generated in China has increased nearly eightfold over the past 40 years [1]. The disposal methods for municipal solid waste typically include landfilling, incineration, and composting [2]. Due to advantages such as reducing waste, being harmless, and recovering energy, incineration has become the preferred option for the treatment of municipal solid waste in an increasing number of Chinese cities [3]. In 2019, the volume of waste treated via incineration in China exceeded that in landfill for the first time. By the end of 2022, the ratio of waste disposal via incineration had reached 80%; the annual treatment volume reached 195 million tons; and the number of operational incineration plants exceeded 600 [1,4].

Incineration generates 1.3–4.5% of fly ash for grate furnaces and 6.5–10.9% for fluidized beds [5], containing heavy metals; soluble salts, especially chlorides; and dioxins, which have been classified as hazardous waste in many countries, including China [6]. Typical methods for the treatment of MSWI-FA mainly include solidification/stabilization–landfill, sintering treatment, and melting treatment [6,7,8,9]. The traditional solidification and landfilling treatment of MSWI-FA not only wastes limited land resources but also poses a high risk of pollution due to the easy of heavy metal leaching [10,11]. Melting treatment offers substantial benefits in terms of reducing waste and being harmless, as well as reusing resources, but it necessitates the development and construction of new furnaces [12] and incurs high operating costs (USD 141–494/ton) in China [13]. Accordingly, many scholars have proposed the use of cement kilns for the co-disposal of MSWI-FA [14,15], and pilot projects have been successfully carried out in cement plants in Suzhou and Beijing, China [16,17]. Due to the relatively low operating cost (USD 113–212/ton) and large disposal capacity of the co-disposal of MSWI-FA in cement kilns [13,16], this disposal approach of MSWI-FA has being included in China’s “National Hazardous Waste List” (2016 and 2021 editions) exemption list [18] and has been widely promoted across China in recent years. This method is considered an effective way for the cement industry to transform and upgrade, with good development prospects [19].

The co-disposal of MSWI-FA in cement kilns involves using the fly ash as a raw material in the cement production process, thus replacing part of the cement raw materials, to achieve the triple purposes of resource recovery, waste disposal, and greenhouse gas emission reduction [20,21]. The main components of MSWI-FA are oxides such as SiO_2_, CaO, Al_2_O_3_, and Fe_2_O_3_, accounting for about 70% of the mass of the fly ash [22,23]. However, MSWI-FA contains a large amount of heavy metal elements [4,24,25], including Cu, Zn, As, Cd, Pb, Cr, and Ni [26,27]. When these elements enter the cement kiln, they may either be incorporated into clinker during the production process or be released into the surrounding environment through flue gas emissions [28,29,30]. Among these heavy metals, Cd and Zn—particularly Cd—deserve special attention, as their melting and boiling points are significantly lower than the temperatures in the cement kiln rotary kiln [31]. A mass balance analysis indicated that almost all the Cd in the raw materials ultimately ends up in cement clinker [32]; therefore, Cd has a risk of leaching into the environment through final building materials [33]. To protect the ecological environment, China has established regulations on the concentration of heavy metals in raw materials, cement clinker, and atmospheric emissions from cement kilns involved in waste co-disposal. For example, the technical specifications for the co-disposal of solid waste in cement kilns (GB 30760-2024) set a reference limit of 1.0 mg/kg for Cd concentration in raw materials fed into the kiln [34].

In addition to heavy metals, chlorine in MSWI-FA is another critical factor affecting its co-processing in cement kilns [35]. Chlorides increase heavy metal leaching [36], promote heavy metal volatilization at high temperatures [37], form toxic compounds such as dioxin under low-temperature conditions outside the kiln [38], and have a negative impact on clinker formation and cement behavior [39]. Therefore, the dechlorination pretreatment of MSWI-FA is a prerequisite for its safe disposal and resource utilization [40]. Pan, J. R., et al. [41] found that the maximum permissible addition of MSWI-FA (1.75%), limited by chlorine content, had no significant impact on cement clinker performance. Wei, Y., et al. [42] demonstrated that CO_2_-aided washing could effectively enhance the removal of chlorine from MSWI-FA, allowing for an increase in the addition of fly ash to the cement kiln co-disposal process to 2–4%. Dechlorination methods for MSWI-FA include water washing, the use of chemical additives, and high-temperature calcination [42,43]. Among these methods, water washing is the most commonly used due to its simplicity, low cost, and high efficiency, achieving an over 90% removal rate for soluble chlorides [44]. Yang, Z., et al. [45] investigated the effects of washing conditions on chlorine and heavy metal removal efficiency from MSWI fly ash (air pollution control residue), achieving only 70% chlorine removal efficiency. To meet the dechlorination requirements of MSWI-FA, multi-stage water washing has typically been employed in recent years [43,46]. The study by Li, M., et al. [47] demonstrated that under lower liquid-to-solid ratio conditions, the three-stage counter-current water washing process achieved a Cl removal efficiency exceeding 99% from MSWI-FA. After washing, the soluble Cl content in the residual fly ash was reduced to below 1%. Several prior studies [43,48] have also demonstrated that multi-stage water washing (e.g., two and three stages) can significantly enhance Cl removal efficiency. However, to date, few studies have reported on the behavior of heavy metal, especially cadmium during the multi-stage water washing of MSWI-FA, and on the related limits for the addition of washed MSWI-FA into cement kilns.

In this study, MSWI-FA samples were collected from five representative Chinese cities. The concentrations of heavy metals (Cu, Zn, Cd, Pb, Cr, and Ni) were analyzed, and a three-stage washing process was investigated under laboratory conditions to evaluate its efficiency in removing chlorides and heavy metals, along with mass loss rates. The maximum allowable addition of washed fly ash in cement kiln co-processing was determined based on heavy metal pollution control requirements. The aim of this study was to verify the possibility for the technological use of ash from the incineration of municipal waste. The findings of this investigation will contribute to promoting the implementation of MSWI-FA co-processing in cement kilns.

## 2. Materials and Methods

### 2.1. Sample Collection

The municipal solid waste incineration fly ash (MSWI-FA) samples were collected from 11 mechanical grate incineration plants across five large representative provinces/municipalities spanning the northern and southern regions in China: Guangdong (#1–2, Guangzhou), Anhui (#3, Hefei), Fujian (#4–5, Fuzhou), Beijing (samples #6–10), and Zhejiang (#11, Hangzhou). All the facilities employed similar combustion technologies and air pollution control systems, including selective non-catalytic reduction (SNCR) for NOx removal, semi-dry/dry scrubbing (SDS/DS) for acid gas neutralization, activated carbon injection (ACI) for heavy metal and dioxin adsorption, and fabric filters (FFs) for particulate collection. Fly ash was sampled at the FF discharge points.

### 2.2. Simulation of the Three-Stage Water Washing Process

**Figure A1:**
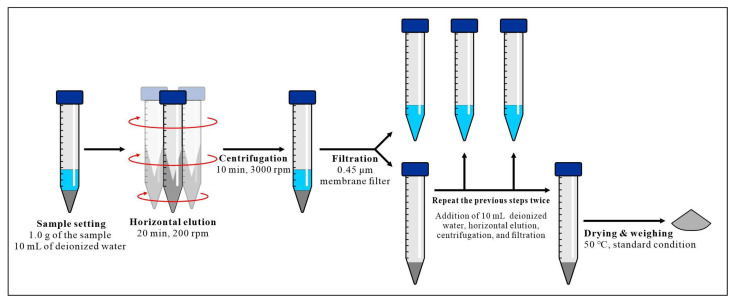
Schematic diagram of the simulated three-stage water washing process for MSWI-FA.

To evaluate the efficiency of the widely used three-stage counter-current washing technique in removing chlorine and heavy metal from MSWI-FA, a simulation of the washing process was conducted as illustrated in Figure A1. The procedure was carried out at a liquid-to-solid ratio of 10 mL/g, with shaking at 25 °C (200 rpm) for 20 min. After each wash, the residue was centrifuged and washed twice more with deionized water following the same steps. Following three washing and centrifugation cycles, the washed fly ash was dried at 50 °C and weighed. The three washing liquids were combined and analyzed for chlorine and heavy metal content. By comparing the chlorine and heavy metal concentrations in the fly ash before and after washing, the changes in chlorine and heavy metal content during the process were determined. The removal ratios were calculated by measuring the total amount of chlorine and heavy metal in the washing liquid and comparing these values with their initial quantities in the raw fly ash.

### 2.3. Sample Testing Methods

The Cu, Zn, As, Cd, Pb, Cr, and Ni contents in the MSWI-FA samples were determined via ICP-MS after acid digestion with HNO_3_ and HF. The process involved weighing 50 mg of the sample, placing it in a polytetrafluoroethylene (PTFE) inner cup, adding 1 mL of concentrated HNO_3_ and HF, sealing the cup in a stainless-steel canister, and allowing digestion to occur at 190 °C for 24 h. After digestion, the solution was evaporated at 120 °C, followed by the addition of 0.5 mL of HNO_3_. After drying, 2 mL of HNO_3_, 2 mL of deionized water, and 1 mL of the rhodium internal standard were added and heated at 140 °C for 5 h. The solution was then cooled, mixed, transferred to a centrifuge tube, diluted to 10 mL, and analyzed via ICP-MS. The samples with the washing liquid were tested directly. Quality control included blank, duplicate, and standard reference samples. The deviation between duplicates was under 10%, and the recovery rate for the heavy metals in standard reference materials (NIST 1633c coal fly ash produced by National Institute of Standards and Technology, U.S. Department of Commerce (Gaithersburg, MD, USA) and BCR 176R municipal solid waste incineration fly ash supplied by Institute for Reference Materials and Measurements of the European Commission (Brussels, Belgium)) ranged from 97% to 108%. The total amount of heavy metals in the washing liquid and washed fly ash from 11 samples ranged from 87.7% to 95.8% of the pre-wash total, confirming the reliability of the results.

The chlorine content in the fly ash was determined based on methods for solid samples, including those for cement (JC/T1073-2008) [49] and coal (GB/T 3558-2014) [50]. First, chlorine was transferred to the liquid phase, excess silver nitrate was added in an acidic medium, and titration was performed with potassium thiocyanate to determine the chlorine content. Then, the amount of chlorine in the washing liquid was measured using the national standard for water (GB 11896-89) [51]. BCR 176R was used as a certified reference material to determine the chlorine (Cl) content in waste incineration fly ash, though a recommended Cl value was not provided. Our measurements of the Cl content in BCR 176R yielded a result of 6.94 ± 0.69% (n = 5). This result is close to the BCR 176R Cl content (7.3%) reported by Lane, D. J., et al. [52], demonstrating that the amount of Cl determined in our waste incineration fly ash is highly reliable.

## 3. Results and Discussion

### 3.1. Heavy Metal and Chlorine Content in Raw Fly Ash

**Figure 1:**
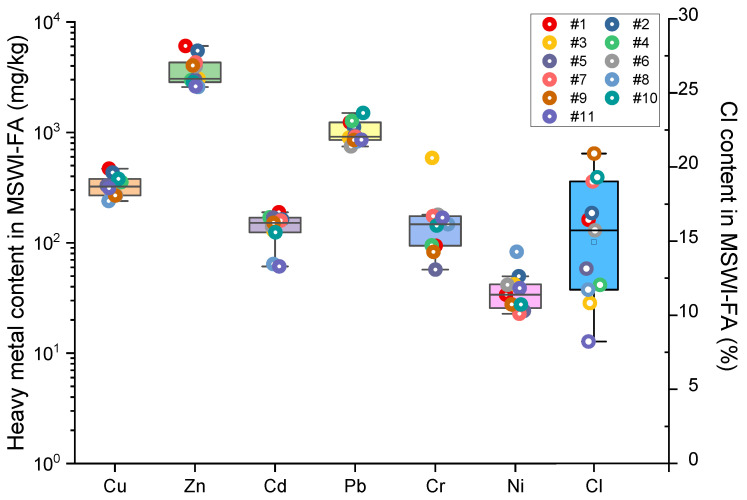
Heavy metals (mg/kg) and Cl (%) content in raw MSWI fly ash.

Figure 1 illustrates the concentrations of heavy metals and chlorine in MSWI-FA. The data reveals that zinc (Zn) has the highest concentration among the heavy metals, while arsenic (As) has the lowest. The concentration of heavy metals in the MSWI-FA is ranked in the following order: Zn (3720 ± 1126 mg/kg) > Pb (1013 ± 223 mg/kg) > Cu (331 ± 69 mg/kg) > Cr (172 ± 137 mg/kg) > Cd (138 ± 40 mg/kg) > Ni (38 ± 16 mg/kg) > As (30.2 ± 17.7 mg/kg). This sequences generally agrees with that from a study analyzing heavy metals in incineration fly ash from waste incineration plants in China [26], as well as with those from other studies [4,53]. Wang et al. [26] conducted a statistical analysis and estimation of the heavy metal contents in waste incineration fly ash (MSWI) in China from 2003 to 2017. Their findings indicated that the arithmetic average concentrations of Cd, Pb, Cr, Zn, Ni, Cu, and As were 116, 1960, 510, 6470, 121, 981, and 158 mg/kg, respectively. In the present study, the concentrations of Pb, Cr, Zn, Ni, Cu, and As were all below these averages, except for the concentration of Cd, which was slightly higher than the reported value. In addition, when compared with the background values of heavy metals in surface soils in China (Cu: 22.6 mg/kg; Zn: 74 mg/kg; As: 11.2 mg/kg; Cd: 0.097 mg/kg; Pb: 26 mg/kg; Cr: 61 mg/kg; and Ni: 26.9 mg/kg) [54] and the risk thresholds for heavy metal contamination in agricultural soils (Cu: 50–200 mg/kg; Zn: 200–300 mg/kg; As: 20–40 mg/kg; Cd: 0.3–0.8 mg/kg; Pb: 70–240 mg/kg; Cr: 150–350 mg/kg; and Ni: 60–190 mg/kg, per GB 15618-2018) [55], as well as the average values of various raw materials in cement factories (Cu: 7.5 mg/kg; Zn: 25 mg/kg; As: 10.1 mg/kg; Cd: 0.37 mg/kg; Pb: 10.65 mg/kg; Cr: 11.1 mg/kg; and Ni: 11.2 mg/kg) [56], waste incineration fly ash was found to be particularly enriched in Cu, Zn, Cd, and Pb, with enrichment factors of 15, 50, 1422, and 39, respectively, compared with the background soil values.

Table 1 presents a comparison of heavy metal contents in MSWI-FA from various studies. It shows that, despite notable regional differences in heavy metal concentrations, zinc (Zn) and lead (Pb) consistently exhibit higher levels, whereas nickel (Ni) and arsenic (As) generally have lower concentrations across different regions. This pattern indicates a relative consistency in the sources of heavy metals in municipal waste. The Cl content ranged from 8.22% to 20.90%, with an average of 14.93 ± 3.84%. This result aligns with previous findings [7,57,58], with the chlorine content in MSWI-FA typically ranging from 1% to 37.3%.

**Figure 2:**
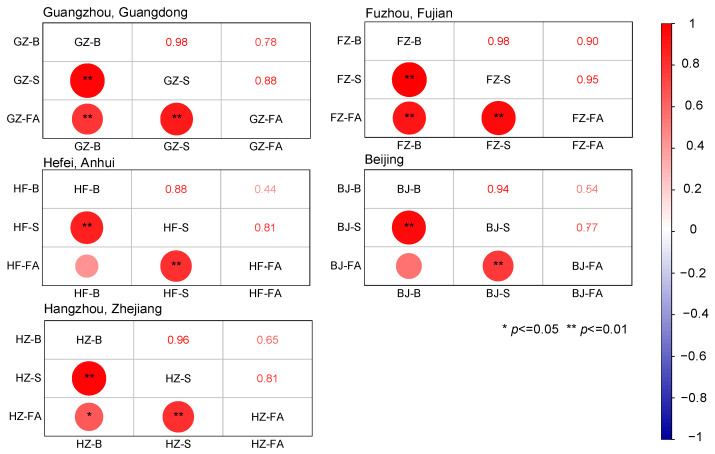
Correlation of heavy metal content in MSWI-FA(FA) with soil background value (B) and surface soil heavy metal content (S) in five typical cities in China.

**Figure A2:**
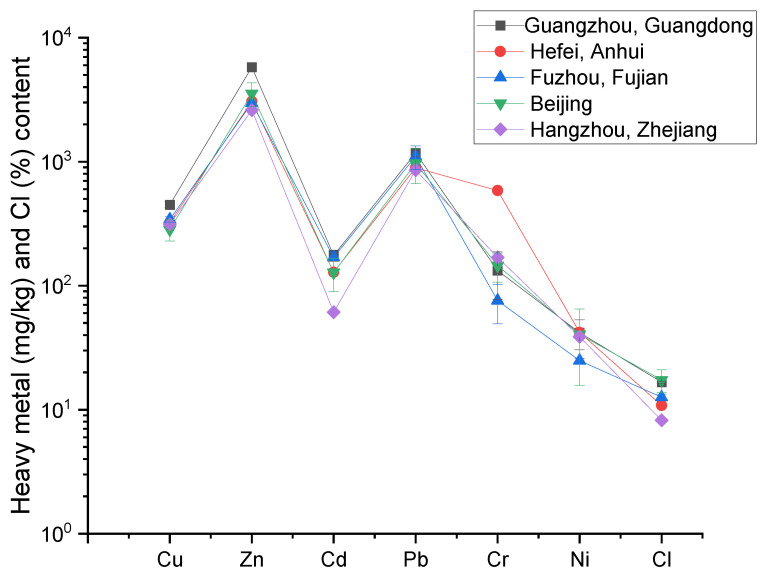
The variations in heavy metal (mg/kg) and Cl (%) content in MSWI-FA in five typical cities in China.

The heavy metal content in waste incineration fly ash also varies considerably by region (Figure A2). Among the five typical cities in China, Guangzhou exhibited the highest levels of Cu, Zn, Cd, and Pb. Hefei had the highest Cr content. Fuzhou recorded the lowest levels of both Cr and Ni. Beijing had the highest Cl content, while Hangzhou showed the lowest levels of Zn, Cd, and Cl in its MSWI-FA. Regional variations in the heavy metal content of MSWI fly ash highlight differences in the physical composition of municipal solid waste between different cities [77]. These disparities may also be affected by the natural background levels of heavy metals in the soils of each region. In 2014, Cheng et al. [78] conducted a systematic study on the background values of heavy metals in soil and the heavy metal content in surface soil across major cities in China. Based on their data, we examined the correlations between soil heavy metal background levels, surface soil heavy metal concentrations, and heavy metal content in MSWI-FA. The results, displayed in Figure 2, show that the heavy metal content in MSWI-FA in all the cities was strongly positively correlated with that in surface soils. Notably, this correlation was stronger with surface soil heavy metal content than that with soil background levels. In particular, the correlation between heavy metal (Zn, Pb, Cu, Cr, Cd, and Ni) content in MSWI-FA and surface soil in all five cities was highly significant (*p* < 0.01). These results indicate that the heavy metal content of surface soil is a key factor influencing the heavy metal content in waste incineration fly ash.

**Figure 3:**
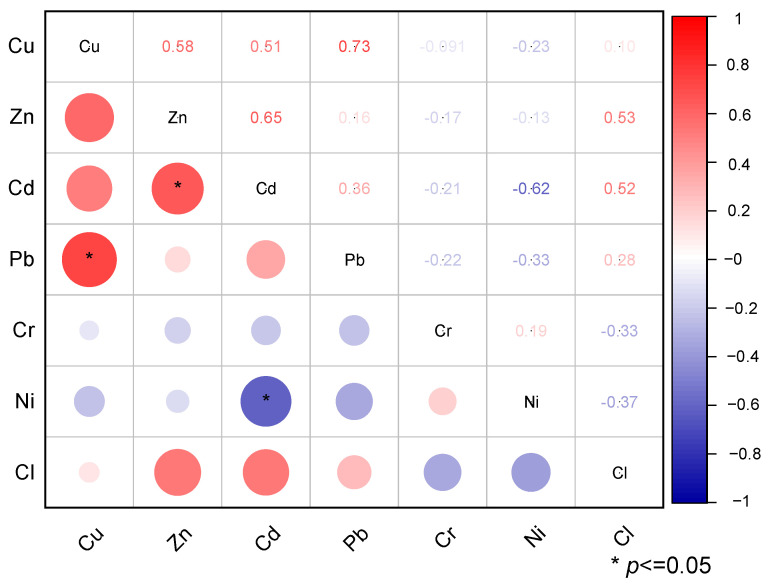
Correlation matrix of heavy metal elements and chlorine in MSWI-FA.

Figure 3 presents the correlation matrix of heavy metal elements and Cl in MSWI-FA. As shown, significant positive correlations are found between Cu and Pb and Zn and Cd, as is a significant negative correlation between Cd and Ni. Additionally, some positive correlations are observed between Cu and Zn, Cu and Cd, and Cl and both Zn and Cd. Cu and Pb in MSWI-FA are commonly sourced from electronic waste, electrical wiring, plumbing materials, and paint. Zn primarily originates from galvanized materials, batteries, and rubber, while Cd is commonly found in Ni-Cd batteries, plastic stabilizers, pigments, and metal plating. Cl often comes from plastics (especially polyvinyl chloride, PVC), industrial waste, and salt-based substances. Zinc and Cd have relatively low boiling points (particularly Cd), making them highly volatile at the high temperatures encountered during incineration. In contrast, Cu and Pb have higher melting and boiling points. During the incineration process, Cl can generate HCl and form volatile chlorides with other volatile metal elements such as Zn and Cd (e.g., ZnCl_2_ and CdCl_2_). The correlations observed among elements in municipal solid waste incineration fly ash can be attributed to the types of waste present and the physicochemical properties of the elements during the incineration process.

### 3.2. Mass Loss of Fly Ash and Removal Rates of Heavy Metal and Chlorine During the Washing Process

**Figure 4:**
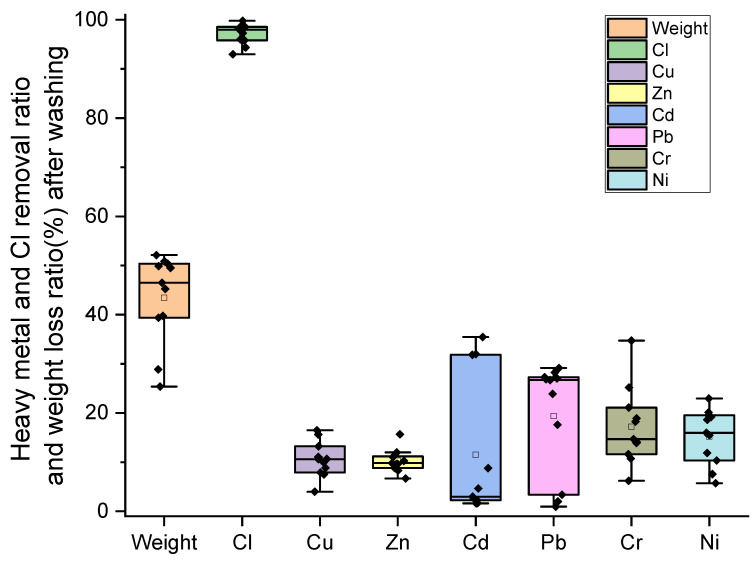
Removal ratio of heavy metal and Cl and weight loss of MSWI-FA during the washing process.

The average mass loss of incineration fly ash caused by the washing process in this study was 43.4 ± 9.2%, ranging from 25% to 52% (Figure 4). In another study, Chang [79] reported that the mass loss of grate furnace fly ash reached up to 40%. The loss rate of grate furnace fly ash in this study is consistent with that in Chang’s research. This is mainly because the fly ash contains a large amount of soluble salts (such as sodium chloride and potassium chloride) and calcium ions.

**Figure 5:**
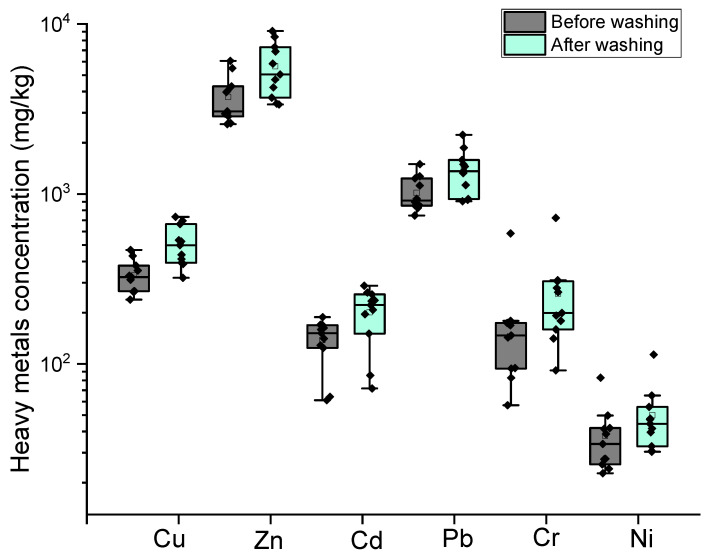
Changes in heavy metal content of MSWI-FA before and after water washing.

The primary purpose of three-stage water washing is to remove chlorine Cl. According to the Code for Design of Cement Plant (GB 50295-2016) [80], the Cl content in cement raw materials should be less than 0.03%. If the only source of Cl in the cement raw material is from MSWI-FA, and the addition ratio is set at 3%, then the Cl content in the fly ash must be less than 1%. As shown in Figure 4, the efficiency of Cl removal by water washing ranged from 93% to 99.8%, with an average removal ratio of 97.1 ± 2.0%. After washing, the Cl content in the residue ranged from 0.02% to 1.16%, with an average of just 0.45 ± 0.32%. These results demonstrate that the three-stage washing process effectively removes Cl from MSWI-FA, making the washed fly ash suitable for use as a raw material in cement production. In addition, although the concentrations of heavy metals increased in the solid residue after water washing (Figure 5), the process still removed a significant portion of soluble heavy metals due to the substantial mass loss of fly ash caused by washing. The proportion of heavy metals lost during the washing process varied significantly among different metals (Figure 4). Pb had the highest removal ratio, with an average of 19.38 ± 10.98%, followed by Cr at 17.22 ± 7.45%, Ni at 15.22 ± 5.35%, Cd at 11.50 ± 13.39%, Cu at 10.53 ± 3.45%, and Zn at 10.28 ± 2.21%.

Figure 5 shows the changes in heavy metal content before and after washing. The figure reveals that the content of all the metal elements increased significantly. Cr showed the highest increase, with an average increase of 60.83 ± 29.43%, while Ni had the smallest increase, at 32.54 ± 15.81%. The order of concentration increase is as follows: Cr > Cu (53.42 ± 20.19%) > Zn (49.90 ± 18.81%) > Cd (43.48 ± 18.90%) > Pb (35.20 ± 18.68%) > Ni. Several previous studies [14,22,81] also demonstrated a significant increase in Cu, Zn, Cd, Pb, and Cr levels in MSWI fly ash after washing. The differences in the content of heavy metals in fly ash before and after water washing mainly depend on two factors: (1) the different amounts of weight lost from the fly ash (dissolved substances such as soluble salts) during the water washing process, and (2) the difference in forms of heavy metals in fly ash and the resultant proportion of heavy metals entering the washing solution.

**Figure 6:**
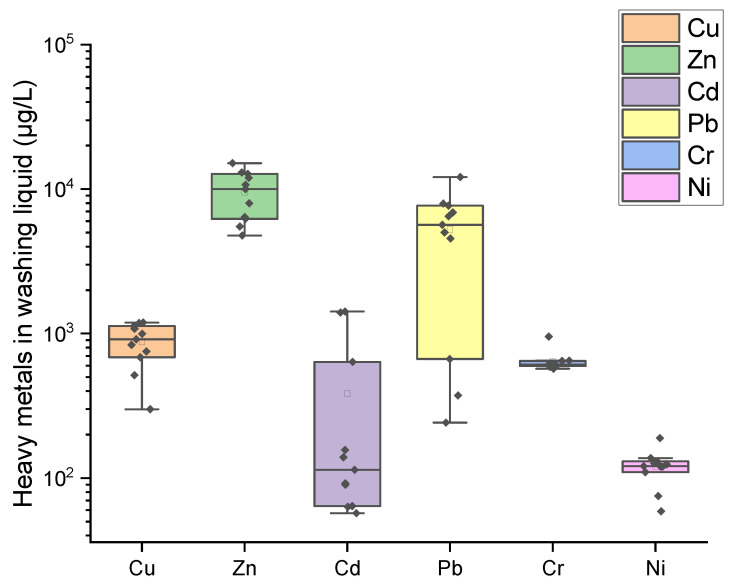
Heavy metal concentration in the washing liquid.

Figure 6 illustrates the concentrations of heavy metals in the aqueous washing solution. The observed concentrations are 299 to 1192 μg/L for Cu, 4768 to 10,004 μg/L for Zn, 57 to 114 μg/L for Cd, 242 to 5661 μg/L for Pb, 571 to 953 μg/L for Cr, and 59 to 121 μg/L for Ni. The discharge standards set by GB 18918-2002 (pollutant discharge standards for urban sewage treatment plants) [82] are 500, 1000, 10, 100, 100, and 50 μg/L for Cu, Zn, Cd, Pb, Cr, and Ni, respectively. Except for Cu, which only exceeds its standard by about 90.9%, all the other heavy metals consistently surpassed their respective limits by several to tens of folds. For example, the average exceedance levels were 1.7 times the standard for Cu, 9.5 times for Zn, 38 times for Cd, 52 times for Pb, 6.4 times for Cr, and 2.4 times for Ni, with Cd and Pb exhibiting the highest levels of exceedance. Particularly noteworthy is that, driven by the need to conserve energy consumption and reduce wastewater treatment volumes, MSWI-FA is typically washed with water at lower liquid-to-solid ratios (<10) [43]. This may result in the heavy metal concentrations in the washing effluent being significantly higher than the values reported in this study. These results underscore the need for rigorous treatment of the washing solution prior to environmental discharge. Without adequate treatment, the heavy metals in the solution may present a serious pollution risk to the surrounding ecosystem. Fortunately, in the vast majority of cement kiln co-disposal projects for incineration fly ash in China, the incineration fly ash washing water is subjected to evaporation–crystallization treatment to obtain chlorides (such as sodium chloride and potassium chloride), and heavy metals are precipitated in a chemical depositing tank [30]. Therefore, the treated wastewater is recycled.

### 3.3. Maximum Allowable Amount of Incineration Fly Ash Addition for Co-Disposal in Cement Kilns

Due to the high heavy metal content introduced by adding fly ash, which may have adverse effects on human health and the environment in cement production and within cement products, the use of waste incineration fly ash in cement kilns needs to be regulated, and guidelines need to be created. To address this, the State Administration for Market Regulation (SAMR) and the National Standardization Administration of China (NSAC) issued the Technical Specification for Co-disposal of Solid Wastes in Cement Kilns (GB/T 30760-2014) in 2014 [83], which was revised in 2024 (GB/T 30760-2024) [34]. This standard specifies the limits for heavy metal content in cement raw materials and clinker, as well as the concentrations of leachable heavy metals in cement clinker. Among the various heavy metals, the limit for cadmium (Cd) is the most stringent (see Table 2), with a maximum allowable concentration of 1 mg/kg in raw materials fed into cement kilns.

Assuming that the heavy metal or Cl content in waste incineration fly ash is C_b_; the addition rate of washed MSWI-FA is R; the average heavy metal or Cl content in the raw materials (excluding fly ash) is C_m_; and the resulting heavy metal or Cl content in the cement raw material with the addition of washed fly ash is C_t_, with a maximum allowable limit C_max_ as specified by the standard GB/T 30760-2024, the relationship can be expressed as follows:

C_t_ = C_b_ × R + C_m_ × (1 − R)

Given that the heavy metal and Cl content in other cement raw materials is low [53], C_m_ can be ignored. Therefore, the maximum allowable addition rate of waste incineration fly ash is R_max_ = C_max_/C_b_, where C_max_ equals the threshold limit of heavy metals and Cl in cement raw materials (mg/kg) stipulated by the standard GB/T 30760-2024, as listed in Table 2. The results are also shown in Table 2.

Table 2 presents the maximum allowable additions of MSWI-FA to cement raw materials, as calculated from the heavy metal content reported by Wang et al. [26] and in the present study. The data reveal that, based on both the arithmetic and geometric means of heavy metal contents, Cd is the primary limiting factor for using MSWI-FA in cement raw meal, with the maximum allowable additions ranging from 0.5% to 1.49%. This is followed by Pb (3.42% to 5.05%), Zn (5.58% to 8.49%), Cl (6.67% to 10%), and Cu (6.6% to 13.18%). Notably, the maximum allowable addition of Ni is much higher, at up to 143.5%. It is important to note that Wang et al.’s data pertain only to the heavy metals in fly ash before washing, whereas metal concentrations increase significantly after washing. Consequently, the actual maximum allowable additions are likely lower than the values calculated using pre-washing data. A previous study [56] indicated that, using MSWI-FA as a raw material in cement production can lead to a 310% increase in Cd input, with only a 0.9% addition. Since most of the Cd in the raw materials of cement plants ultimately ends up in cement clinker [32], special attention must be paid to the disposal of such cements, for example, heavy metal-laden cements cannot be used to construct buildings for human habitation or buildings in ecologically sensitive areas, and a thorough evaluation of their life cycle is essential [84]. In this study, the heavy metal content in other raw materials used in cement production, including Cd, is not taken into account, and the heavy metal content in waste incineration fly ash can vary significantly depending on the source; therefore, limiting the addition of MSWI-FA to less than 0.5% in China is advisable.

## 4. Conclusions

This study systematically evaluated the feasibility and environmental implications of co-disposing municipal solid waste incineration fly ash (MSWI-FA) in cement kilns through a three-stage water washing pretreatment. The research addresses critical challenges in managing hazardous MSWI-FA, focusing on chloride removal efficiency, heavy metal behavior, and compliance with regulatory standards. The key findings and their implications are summarized as follows:

(1) The heavy metal concentrations in MSWI-FA varied significantly across the five Chinese cities, with Zn (3720 ± 1126 mg/kg), Pb (1013 ± 223 mg/kg), and Cu (331 ± 69 mg/kg) being the most abundant. These variations were strongly correlated with the heavy metal levels in the regional surface soils (*p* < 0.01), highlighting the influence of local soil contamination. Elemental correlations (e.g., Cu–Pb synergy, Zn–Cd association, and Cd–Ni antagonism) further underscored shared waste sources (e.g., electronics, batteries, and PVC plastics) and volatilization dynamics during incineration.
(2) The three-stage counter-current washing process demonstrated exceptional efficiency in chloride removal, achieving an average reduction of 97.1 ± 2.0%, with residual Cl content reduced to 0.45 ± 0.32%. This meets the stringent Cl limit (<1%) required for cement raw materials, enabling MSWI-FA to serve as a viable substitute for traditional raw materials. However, the process exhibited limited effectiveness in removing heavy metals, with removal efficiencies ranging from 10.28% (Zn) to 19.38% (Pb). Notably, the process caused a substantial mass loss (43.4 ± 9.2%) due to the dissolution of soluble salts (e.g., NaCl and KCl), which paradoxically increased the heavy metal concentrations in the residue by 32.5% (Ni) to 60.8% (Cr). This concentration effect exacerbates environmental risks if the washed ash is improperly managed.
(3) Despite successful Cl removal, the washing process generated effluents with heavy metal concentrations exceeding China’s municipal wastewater discharge standards (GB 18918-2002) by up to 52-fold for Pb and 38-fold for Cd. This poses severe contamination risks if untreated effluents are released into ecosystems. Furthermore, the enrichment of Cd in washed ash emerged as the most critical constraint for co-disposal in cement kilns. Calculations based on China’s GB/T 30760-2024 standard revealed that Cd’s strict limit (1 mg/kg in raw materials) restricts the maximum addition of washed MSWI-FA to ≤0.5% in cement production.

In conclusion, this work underscores the potential of cement kiln co-processing as a sustainable pathway for MSWI-FA management in China. However, its success hinges on balancing resource recovery with rigorous environmental safeguards. By addressing technical, regulatory, and regional challenges, this approach can contribute to circular economy goals while mitigating risks to human and ecological health.

## Figures and Tables

**Table 1:** Heavy metal content of MSWI-FA for different regions.

Area	Heavy Metal						References
	Cu	Zn	Cd	Pb	Cr	Ni	
China	331 ± 69	3720 ± 1126	138 ± 40	1013 ± 223	172 ± 137	38 ± 16	This study
China	981	6470	116	1960	510	121	[26]
China	1141 ± 323.8	14,092 ± 5120	170 ± 29.7	2701 ± 605	554 ± 77	154 ± 44.7	[59]
China	3315	5692	-	933	423	-	[60]
China	1385.0	7079.0	189.2	5253.0	364.0	228.2	[53]
Hangzhou, China	500	19,880	350	5480	250	90	[61]
Taiwan, China	1600 ± 20	1000	83 ± 1	2500 ± 10	230 ± 30	-	[22]
Taiwan, China	1100 ± 100	8900 ± 300	300	1600 ± 100	500	-	[40]
Shanghai, China	199 ± 1	1267 ± 61	29 ± 0	852 ± 5	72 ± 2	-	[62]
Wuhan, China	756.15	2683.93	-	1377.12	93.76	-	[63]
Shanghai, China	603.34	3487.23	62.33	2369.31	132.91	24.66	[64]
Liaoning, China	924 ± 46	7332 ± 219	-	2703 ± 81	549 ± 12	-	[65]
Eastern China	1587 ± 1138	5072 ± 1536	62 ± 38	1259 ± 522	339 ± 166	112 ± 63	[66]
Shandong, China	377 ± 14	4344 ± 311	122 ± 17	1251 ± 80	119 ± 29	25 ± 10	[67]
Beijing, China	394	3263	70	642	26	120	[15]
Shanghai, China	603.4 ± 31.5	3497.7 ± 278.6	64.8 ± 5.1	2375.2 ± 186.6	132.9 ± 10.8	23.6 ± 1.4	[68]
Dalian, China	740	6368	282	2249	55	12	[69]
Beijing, China	702.2	4959	108.1	1111.2	49.7	-	[58]
Beijing, China	412.3	4240.6	126.9	983.1	278.3	-	[57]
Japan	460 ± 252	4300 ± 2621	68 ± 42	1480 ± 1370	120 ± 36	15 ± 5	[70]
Japan	-	-	110	-	260	-	[14]
Japan	423.6 ± 2.1	423.6 ± 2.1	57.3 ± 0.20	1623 ± 17	240.9 ± 2.3	-	[71]
Korea	1087 ± 575	6700 ± 1308	303 ± 97	2967 ± 651	141 ± 80	37 ± 19	[70]
USA	630	16,000	260	5600	140	30	[72]
Australia	702 ± 105	9750 ± 1660	140 ± 35	1730 ± 340	250 ± 9	54 ± 4	[73]
Italy	881	14,400	-	5076	1459	88	[74]
Switzerland	3006 ± 775	46,710 ± 19,242	282 ± 65	10,234 ± 1591	604 ± 103	-	[75]
Finland	1955 ± 1903	12,433 ± 6178	143 ± 107	2488 ± 1928	561 ± 111	133 ± 43	[52]
Northern Vietnam	1508	2809	20.95	2169	665.8	-	[76]

**Table 2:** The calculated maximum dosage ratio for co-disposal of MSWI-FA in cement kilns (based on raw materials).

Heavy Metal	TL-CRM	HM-FA (AM) [26]	MAR	HM-FA (GM) [26]	MAR	HM-FA (AM)	MAR	HM-FA (GM)	MAR
Cu	65	981	6.60	664	9.79	590.5	11.0	493	13.18
Zn	361	6470	5.58	4250	8.49	5631.0	6.41	5310	6.80
Cd	1	116	0.86	67.3	1.49	201.2	0.50	186	0.54
Pb	67	1960	3.42	1420	4.72	1381.5	4.85	1328	5.05
Cr	98	510	19.22	253	38.74	258.8	37.88	224	43.8
Ni	66	121	54.55	70.2	94.01	49.9	132.26	46	143.5
Cl	0.03	-	-	-	-	0.45	6.76	0.30	10

TL-CRM: threshold limit in cement raw materials (mg/kg for heavy metals, % for Cl); HM-FA: heavy metal content in MWSI-FA (mg/kg); MAR: maximum addition ratio (%); AM: arithmetic mean; GM: geometric mean.

## Data Availability

The raw data supporting the conclusions of this article will be made available by the authors on request.
